# Evolutionary Patterns of Modularity in the Linkage Systems of the Skull in Wrasses and Parrotfishes

**DOI:** 10.1093/iob/obad035

**Published:** 2023-09-26

**Authors:** S M Gartner, O Larouche, K M Evans, M W Westneat

**Affiliations:** Organismal Biology and Anatomy Department, University of Chicago, Chicago, IL 60637, USA; Department of Biology and Biochemistry, University of Houston, Houston, TX 77204, USA; Department of Biosciences, Rice University, Houston, TX 77005, USA; Organismal Biology and Anatomy Department, University of Chicago, Chicago, IL 60637, USA

## Abstract

The concept of modularity is fundamental to understanding the evolvability of morphological structures and is considered a central framework for the exploration of functionally and developmentally related subsets of anatomical traits. In this study, we explored evolutionary patterns of modularity and integration in the 4-bar linkage biomechanical system of the skull in the fish family Labridae (wrasses and parrotfishes). We measured evolutionary modularity and rates of shape diversification of the skull partitions of three biomechanical 4-bar linkage systems using 205 species of wrasses (family: Labridae) and a three-dimensional geometric morphometrics data set of 200 coordinates. We found support for a two-module hypothesis on the family level that identifies the bones associated with the three linkages as being a module independent from a module formed by the remainder of the skull (neurocranium, nasals, premaxilla, and pharyngeal jaws). We tested the patterns of skull modularity for four tribes in wrasses: hypsigenyines, julidines, cheilines, and scarines. The hypsigenyine and julidine groups showed the same two-module hypothesis for Labridae, whereas cheilines supported a four-module hypothesis with the three linkages as independent modules relative to the remainder of the skull. Scarines showed increased modularization of skull elements, where each bone is its own module. Diversification rates of modules show that linkage modules have evolved at a faster net rate of shape change than the remainder of the skull, with cheilines and scarines exhibiting the highest rate of evolutionary shape change. We developed a metric of linkage planarity and found the oral jaw linkage system to exhibit high planarity, while the rest position of the hyoid linkage system exhibited increased three dimensionality. This study shows a strong link between phenotypic evolution and biomechanical systems, with modularity influencing rates of shape change in the evolution of the wrasse skull.

## Introduction

Morphological and biomechanical traits often have varying levels of interrelationships with other such traits that can reflect their level of evolutionary independence or their tendency to coevolve as tightly associated units or modules (Wagner 1996). The variation in independence and arrangement between traits can be analyzed using the concepts of modularity and integration. Prior modularity research has often shown patterns of covariation to be unevenly distributed in a system, where some traits show higher correlation, whereas others show higher independence (Cheverud 1982, 1995; Olson and Miller 1999; Albertson et al. 2005; Klingenberg 2008; Goswami and Polly 2010; Parsons et al. 2011, 2012; Goswami et al. 2019). These patterns of modularity can result in sets of semi-independent traits that can evolve at different evolutionary rates or respond to evolutionary or biomechanical forces differently, potentially leading to new functional or structural innovations (i.e., mosaic evolution). Identifying these patterns over a large phylogenetic sample can hint at the developmental, functional, or environmental influences driving the evolution of a system (Cheverud 1982, 1995; Olson and Miller 1999; Klingenberg 2008; Larouche et al. 2018; Adams and Collyer 2019; Bardua et al. 2019; Churchill et al. 2019; Goswami et al. 2019). Modularity analyses applied to complex anatomical systems in a large and diverse group of species can reveal macroevolutionary patterns in a group and help understand the impacts of change in complex functional systems.

**Fig. 1:**
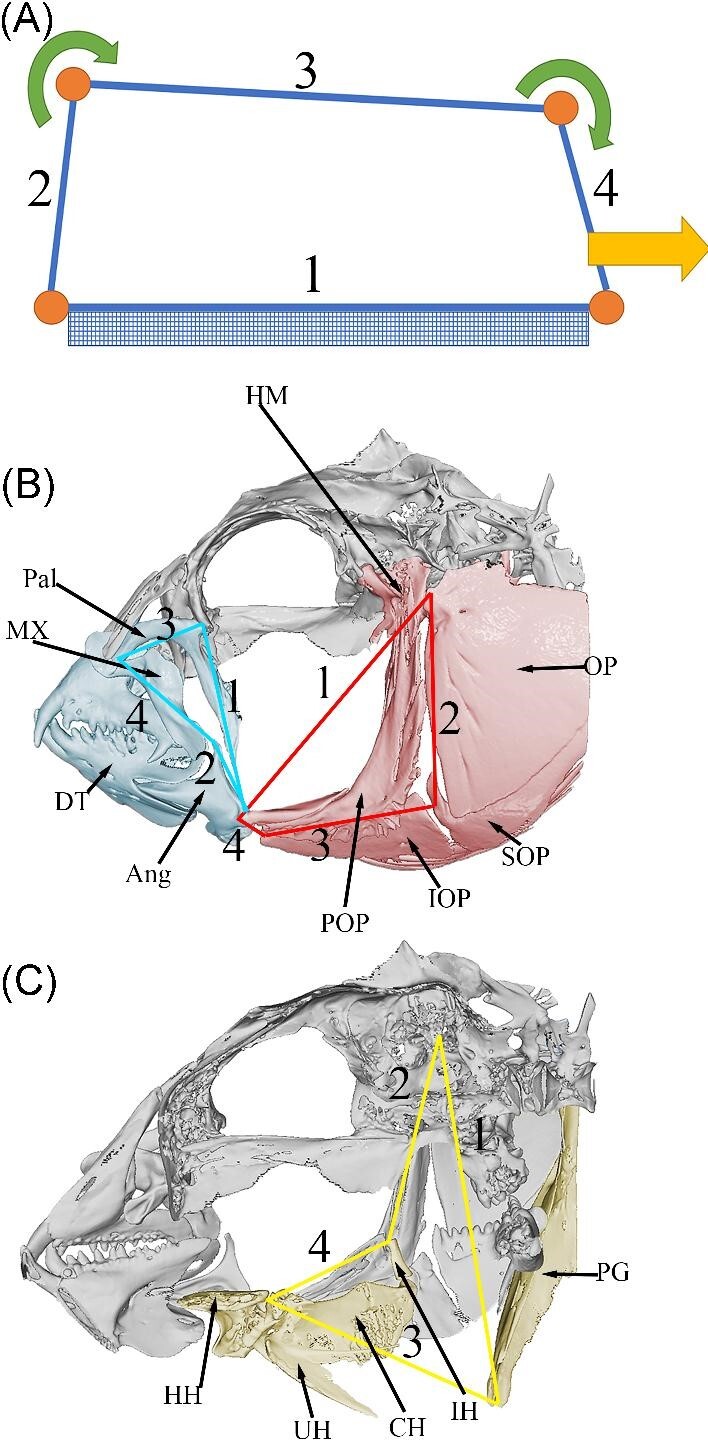
**(A)** Basic schematic of the 4-bar linkage. 1 represents the fixed link, 2 input link, 3 coupler link, and 4 output link. These letterings correspond to (**B)** and (**C)**. (**B)** Lateral view of *Halichoeres argus* with the anterior jaw (blue) linkage with the associated bones highlighted in blue and opercular (red) linkage with the associated bones highlighted in red. (**C)** lateral view of the inside of *H. argus* with the hyoid (yellow) linkage with the bones involved with the linkage system highlighted in yellow. Abbreviations: Pal—palatine; PX—premaxilla; DT—dentary; Ang—angular; POP—preoperculum; OP—operculum; SOP—suboperculum; IOP—interoperculum; IH—interhyal; PG—pectoral girdle; CH—ceratohyal; UH—urohyal; HH—hypohyal; HM—hyomandibula.

Fish-feeding systems are among the most diverse morphological and kinetic structures in vertebrates. The teleost fish skull is a highly complex structure composed of multiple bony elements connected by soft tissues that function in a diversity of ways to successfully feed on prey (Liem 1970; Anker 1974; Barel 1982; Lauder 1982; Muller 1989; Westneat 2006). The functional systems of the skull in most teleost fish operate as biomechanical levers and linkages that control the movements of bones and coevolve with the morphological structures (Westneat 1990; Wainwright et al. 2004). The three major 4-bar linkage systems in the perciform skull are the anterior jaw linkage, the opercular linkage, and the hyoid linkage (Westneat 1990). These linkages have been modeled as a 4-bar linkage system using mechanical engineering principles (Anker 1974; Muller 1989; Westneat 1990; Olsen and Westneat 2016), where the movement of three mobile links connected by rotating joints has computationally defined motions relative to a fourth fixed link (Fig. 1). The linked nature of these mechanical systems suggests the hypothesis that each linkage may be tightly integrated and that the three linkages may show a high level of integration with one another. Alternatively, it has been shown that there is a frequent phylogenetic divergence among close relatives in these systems, leading to a broader pattern of convergence across groups (Westneat et al. 2005), suggesting the alternative hypothesis that functional diversity may be driven by independent evolution of linkage systems.

Teleost skulls have been the subject of multiple modularity studies (Evans et al. 2017, 2019, 2022; Ornelas-García et al. 2017; Baumgart and Anderson 2018; Larouche et al. 2018, 2022). Recent work in the labrid fishes (Larouche et al. 2022) found functional hypotheses, instead of developmental hypotheses, to be more strongly supported in a modularity analysis of the skull. This work also found that the best-fitting functional hypotheses exhibited patterns in which traits with similar functions (i.e., oral jaws and pharyngeal jaws) were more tightly integrated than traits that share developmental origins (i.e., maxilla and premaxilla). These recent studies have revealed patterns of modularity based on developmental and functional systems in the teleost skull, yet a central question remaining is the degree to which the biomechanical linkage systems of the skull are tightly integrated as modules. The linkage systems all share a similar function in that they help to move the skull during different behaviors (i.e., feeding, breathing, and social communication). Mainly, the anterior jaw linkage system functions to move the oral jaws, the opercular linkage system moves the opercular series and the lower jaw, and the hyoid linkage system moves the hyoid apparatus and the lower jaw (Fig. 1). This leads to the question: Does having a shared function lead to higher covariation among traits? Are the 4-bar linkage systems modular at an evolutionary level? In other words, do the skulls parse out into modules described by the 4-bar linkage systems across lineages? Here, we further investigate the functional trends that govern the modularity and integration of the fish skull, testing the level of integration in and among the modules defined by the biomechanical 4-bar linkages of the teleost skull. We try to further understand whether covariation of bone shape is a signal of the functional modularity of the linkage systems (e.g., whether the linkage systems are functionally transforming together).

The 4-bar linkage systems in fishes have been modeled as two-dimensional (2D; Anker 1974; Muller 1989; Westneat 1990) and three-dimensional (3D; Olsen and Westneat 2016; Olsen et al. 2017) structures, with recent work revealing the importance of accounting for 3D linkage structure when present. In addition, modularity was shown to have a relationship with the kinematic transmission (KT) of 3D 4-bar linkage systems in salmon (Baumgart and Anderson 2018). Our 3D geometric morphometrics of linkage structures allows us to address the question of planarity and deviation from planarity across a large sample of labrid fish. Here, we test the planarity, or two dimensionality, of 4-bar linkage systems to understand whether three dimensionality, or lack thereof, affects the modularity of the system.

Wrasses and parrotfishes (family: Labridae) are a group of coral reef fishes with a wide diversity in skull shape and have a well-resolved phylogeny (Aiello et al. 2017; Larouche et al. 2022). This group of fish represents the second largest marine fish family and has been shown to have a wide diversity in skull shape and function, with the amount of diversity in each tribe showing divergence in lever and linkage mechanisms (Wainwright et al. 2004; Westneat et al. 2005). These findings provide an opportunity to investigate the family-wide and tribe-specific evolutionary influences on the skull and associated skeletal elements. Using this diversity, we seek to quantify the relationship between the 4-bar linkage systems and skull morphology over evolutionary time. Thus, the main objectives of this study were to investigate the modularity patterns seen across wrasse linkage systems and in four main wrasse tribes (hypsigenyines, julidines, cheilines, and scarines) and to test whether the best-fitting hypotheses of modularity led to differences in phylogenetic rates of linkage diversification. In addition, because 4-bar linkages are typically modeled as planar transmission systems, we set out to quantify the three dimensionality of the linkage systems by developing a metric of linkage planarity to understand whether modularity is affected by the relative planarity versus three dimensionality of these mechanical systems.

## Methods

### Phylogeny

**Fig. 2:**
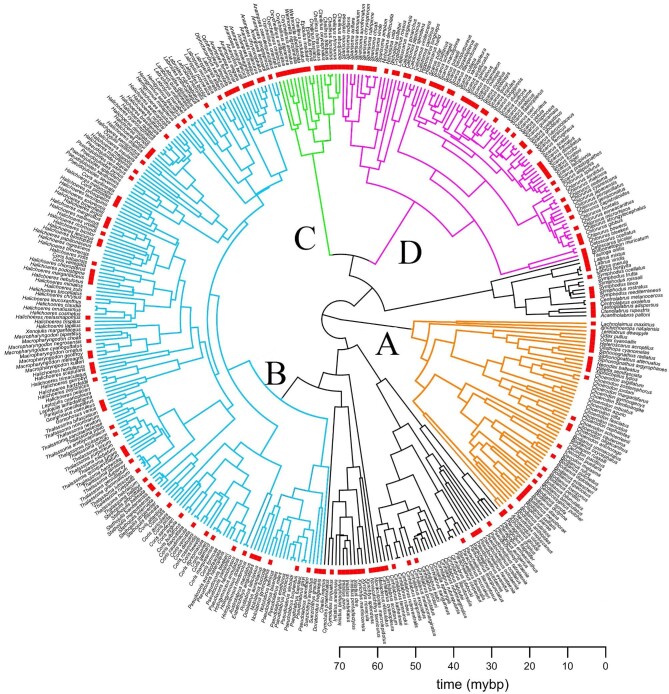
Time-calibrated tree of the Labridae (410 species) from Larouche et al. 2022 with red bars indicating presence of the 205 wrasse species in our data set while colored branches indicate the tribes we analyzed further in the modularity study: (**A)** hypsigenyines highlighted in orange, (**B)** julidines highlighted in blue, (**C)** cheilines highlighted in green, and (**D)** scarines highlighted in pink. A scale bar is provided on the bottom right in million years per before present (mybp).

To provide a framework for modularity testing, we used a recently published phylogenetic analysis of 410 species of labrid fishes (Larouche et al. 2022) (Fig. 2). This tree provides an updated topology and time-tree branch lengths for the family, used in our prior work to explore the tempo and mode of skull modules. The phylogeny was built as a subset of a larger in-progress study of 550 species using a set of 12 genes, both mitochondrial and nuclear, accumulated by a series of recent studies (Westneat and Alfaro 2005; Smith et al. 2008; Aiello et al. 2017) and analyzed in BEAST using the same fossil calibration framework as the recent phylogenomic analysis of the Labridae (Hughes et al. 2022). The resulting time-calibrated Bayesian inference tree was then pruned down to the 205 taxa for which we collected morphometric data using the *drop.tip* function in the R package *ape* (Paradis et al. 2004).

### Geometric morphometrics

We quantified 3D skull shape across 205 labrid species using microcomputed tomography (*μ*CT) scans (previously detailed in Larouche et al. 2022 and Evans et al. 2022). We scanned one individual for each species. Specimens scanned were all adults and free of any sex-specific morphologies (e.g., the hump on the male Napoleon wrasse). Specimens were selected in an appropriate size range for scanning and with jaws closed when available. We calculated jaw gape as a proportion of skull length (Supplementary Table 1) to assess whether jaws were partially open due to the specimen position when preserved. We found about 20 specimens with greater than 10% jaw gape, including several *Anampses* but no cheilines or scarines. To explore the possible influence of these preservational artifacts, we conducted sensitivity analyses by analyzing modularity and evolutionary patterns both with and without those species included. We found that the presence or absence of specimens with their mouths partially open did not impact the modularity or evolutionary rate analyses. The specimens were primarily from the ichthyology collection from the Field Museum of Natural History (FMNH), Chicago, IL. Additional specimens came from the Academy of Natural Sciences of Drexel University (ANSP), the American Museum of Natural History (AMNH), the Australian Museum (AM), the Bell Museum of Natural History (JFBM), the Bernice Pauahi Bishop Museum (BPBM), the Burke Museum of Natural History and Culture (UWFC), the National Museum of Natural History (USNM), and the Natural History Museum (BMNH) (Supplementary Table 2). Specimens were scanned at the University of Chicago, University of Minnesota, and University of Washington Friday Harbor Laboratories as a part of the scanAllFishes and oVert initiatives.

Scans were segmented in Amira v2.0.0 (Thermo Fisher Scientific, Waltham, MA) to isolate the skull and remove scales and debris. We followed the landmarking methods described in Larouche et al. (2022). However, we added three landmarks that describe points along the pectoral girdle (Supplementary Table 3). Briefly, the isolated skulls were converted into 3D meshes and imported into Checkpoint (Stratovan, Davis, CA). Shape variation was described by 200 3D points, 83 landmarks, and 117 semi-landmarks (Supplementary Fig. 1) that adequately sampled the pharyngeal jaws, oral jaws, neurocranium, nasals, hyomandibula, operculum, hyoid apparatus, and pectoral girdle (Supplementary Table 3 for descriptions of each landmark). Points were solely placed on the left side of the skull.

Due to our interest on the linkage systems, and their essential reliance on positioning, we used a General Procrustes Analysis (GPA) (Fig. 2) of the entire composition where the landmark configurations are first centered at their origin (i.e., the centroid), then scaled to unit centroid size and, finally, optimally rotated using an iterative process to minimize the summed squared distances between homologous landmarks (Rohlf and Slice 1990; Zelditch et al. 2012). For the semi-landmarks, the positions were optimized using the criterion of minimizing the Procrustes chord distance between the reference and target specimens. We chose this method due to the alternative criterion of minimum bending energy being shown to potentially introduce spurious spatial autocorrelations among sliding semi-landmarks (Cardini et al. 2019).

### Phylomorphospace

**Fig. 3:**
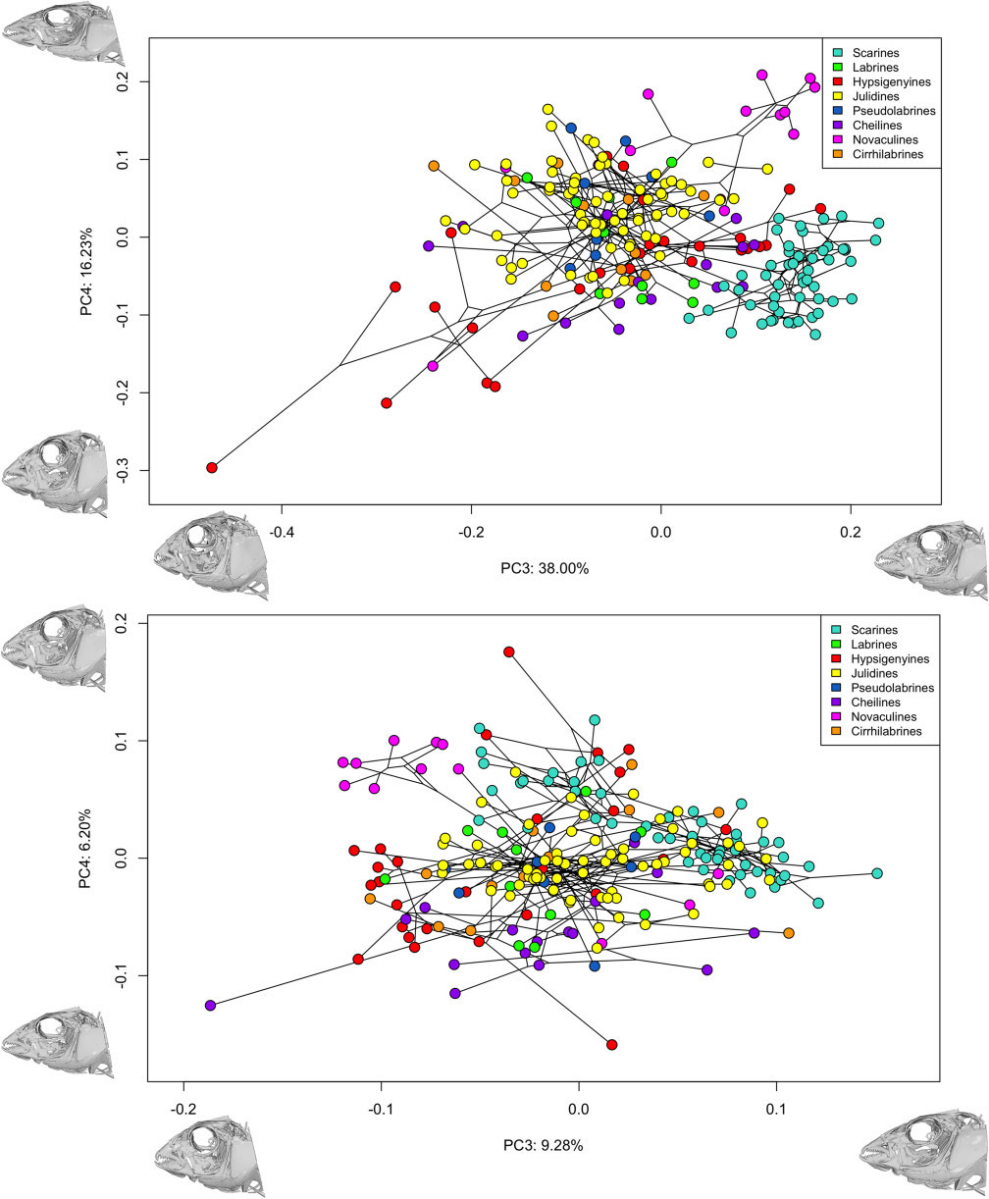
Phylomorphospace with the first two PCs from a PCA of 205 wrasse specimens. 3D mesh insets illustrate changes in landmark positions between the species with the mean shape and the minimum or maximum values along both axes.

**Fig. 4:**
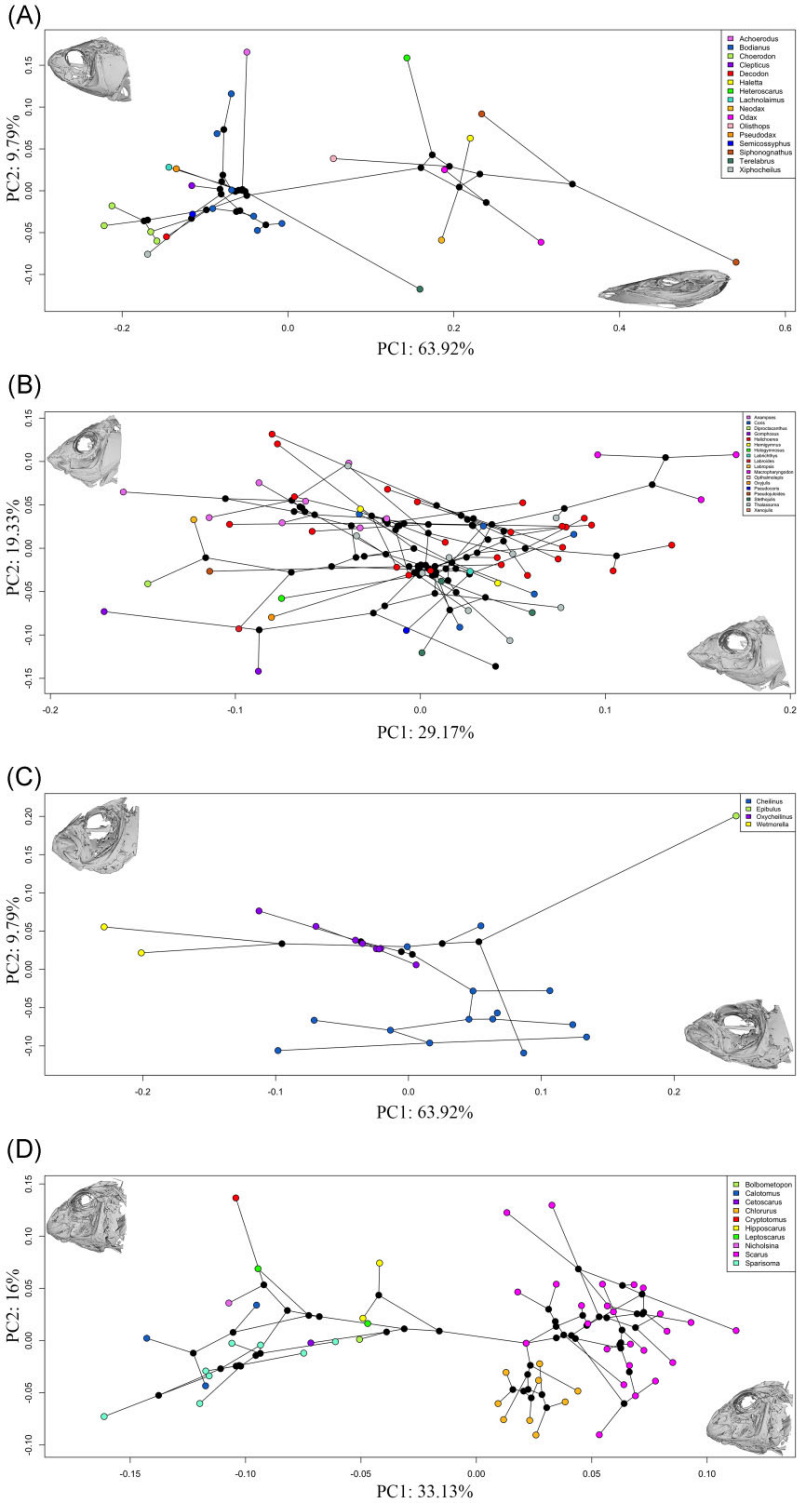
Phylomorphospaces of four Labrid tribes: (**A)** hypsigenyines, (**B)** julidines, (**C)** cheilines, and (**D)** scarines. 3D mesh insets illustrate changes in landmark positions between the species with the lowest and highest variation along both axes.

To explore the phylogenetic patterns of 3D skull morphometrics, and to identify regions of interest, we used a phylomorphospace approach (Sidlauskas 2008). Principal component analysis (PCA) was used as an exploratory method to describe major axes of variation in the skull of the Labridae. Major axes of shape variation were visualized by plotting shape changes between the species with the most extreme scores along each PC axis that captured the most shape variation. We visualized the phylomorphospace using R (RStudio 2012) in the package *phytools* using the function *phylomorphospace* (Revell 2012). We plotted the entire Labridae family (Fig. 3) as well as the tribes of interest (Fig. 4) to visualize the geometric patterns of change along the phylogeny in and across tribes.

### Modularity tests

Thirteen modularity hypotheses were created and tested, determined by the bones directly connected in the 4-bar linkage systems (Table 1). We tested whether the linkage systems were three separate modules affecting different parts of the skull or one module having a shared function of skull movement for the entire family Labridae. The linkages were separated by the bones they interact with the most, with the anterior jaw system including the maxilla, premaxilla, dentary, angular, and palatine; the opercular system including the opercular series, suspensorium, and hyomandibula; and the hyoid system including the hypohyal, ceratohyal, urohyal, and pectoral girdle. The first hypothesis was a control that treated each bone as a separate module to test whether there is a pattern of modularity and integration in the system. Half of the hypotheses treat the premaxilla as a part of the anterior jaw linkage system, while the other half of the hypotheses treat the premaxilla as a separate module from the linkage system due to the premaxilla not being directly associated with the links, despite its motion being directly attributed to the anterior jaw linkage system. The hypotheses also varied in how many modules remained after the linkage systems were placed in modules (see Table 1 for details). We recognize that the opercular 4-bar linkage system has a point on the lower jaw. However, because the lower jaw is more influenced by the anterior jaw 4-bar linkage, we included the lower jaw in the anterior jaw linkage module only.

**Table 1:** Description of all 13 hypotheses tested with the number of modules each hypothesis is testing

Hypothesis	Description of modules	Number of modules
H1	Premaxilla + maxilla + dentary + articular + neurocranium + nasals + upper pharyngeal jaw + lower pharyngeal jaw + ceratohyal + urohyal + hyomandibula + opercular series + palatine + pectoral girdle + hypohyal + suspensorium	14
H2	(Angular * dentary * ceratohyal * opercular series * palatine * maxilla * suspensorium * pectoral girdle * hypohyal * urohyal * hyomandibula * premaxilla) + (nasals * neurocranium * upper pharyngeal jaw * lower pharyngeal jaw)	2
H3	(Angular * dentary * ceratohyal * opercular series * palatine * maxilla * suspensorium * pectoral girdle * hypohyal * urohyal * hyomandibula) + (premaxilla * nasals * neurocranium * upper pharyngeal jaw * lower pharyngeal jaw)	2
H4	(Angular * dentary * palatine * maxilla * premaxilla) + (ceratohyal * hypohyal * urohyal * pectoral girdle) + (opercular series * suspensorium * hyomandibula) + (nasals * neurocranium * upper pharyngeal jaws * lower pharyngeal jaws)	4
H5	(Angular * dentary * palatine * maxilla) + (ceratohyal * hypohyal * urohyal * pectoral girdle) + (opercular series * suspensorium * hyomandibula) + (premaxilla * nasals * neurocranium * upper pharyngeal jaws * lower pharyngeal jaws)	4
H6	(Angular * dentary * ceratohyal * opercular series * palatine * maxilla * suspensorium * pectoral girdle * hypohyal * urohyal * hyomandibula * premaxilla) + nasals + neurocranium + upper pharyngeal jaw + lower pharyngeal jaw)	4
H7	(Angular * dentary * ceratohyal * opercular series * palatine * maxilla * suspensorium * pectoral girdle * hypohyal * urohyal * hyomandibula) + premaxilla + nasals + neurocranium + upper pharyngeal jaw + lower pharyngeal jaw	5
H8	(Angular * dentary * palatine * maxilla * premaxilla) + (ceratohyal * hypohyal * urohyal * pectoral girdle) + (opercular series * suspensorium * hyomandibula) + nasals + neurocranium + upper pharyngeal jaws + lower pharyngeal jaws	7
H9	(Angular * dentary * palatine * maxilla) + (ceratohyal * hypohyal * urohyal * pectoral girdle) + (opercular series * suspensorium * hyomandibula) + premaxilla + nasals + neurocranium + upper pharyngeal jaws + lower pharyngeal jaws	8
H10	(Angular * dentary * ceratohyal * opercular series * palatine * maxilla * suspensorium * pectoral girdle * hypohyal * urohyal * hyomandibula * premaxilla) + (nasals * neurocranium) + (upper pharyngeal jaw * lower pharyngeal jaw)	3
H11	(Angular * dentary * ceratohyal * opercular series * palatine * maxilla * suspensorium * pectoral girdle * hypohyal * urohyal * hyomandibula) + premaxilla + (nasals * neurocranium) + (upper pharyngeal jaw * lower pharyngeal jaw)	4
H12	(Angular * dentary * palatine * maxilla * premaxilla) + (ceratohyal * hypohyal * urohyal * pectoral girdle) + (opercular series * suspensorium * hyomandibula) + (nasals * neurocranium) + (upper pharyngeal jaws * lower pharyngeal jaws)	5
H13	(Angular * dentary * palatine * maxilla) + (ceratohyal * hypohyal * urohyal * pectoral girdle) + (opercular series * suspensorium * hyomandibula) + premaxilla + (nasals * neurocranium) * (upper pharyngeal jaws * lower pharyngeal jaws)	6

*Note*: Parentheses designate separate modules, plus signs represent separate modules, and * represent covariation between modules.

To test patterns of modularity in the tribes, we repeated our analyses of modularity in four tribes that had a large enough sample size of species. The tribes included were hypsigenyines (hogfishes, tuskfishes, and relatives), julidines (crown tribe of many genera), cheilines (maori wrasses), and scarines (parrotfishes).

Hypotheses of evolutionary modularity were compared using the effect size of the covariance ratio (CR), one of the most widely used methods to analyze modularity. The CR is a measure of the relative strengths of associations among partitions of landmarks versus associations in these subsets (Adams and Collyer 2019). CRs were computed with the function *phylo.modularity*, and the effect sizes were compared using the *compare.CR* function; both functions are implemented in the R package *geomorph* (Adams et al. 2022).

We also analyzed the modularity hypotheses using the distance matrix method, which produces a correlation matrix between the shape partitions of landmark subsets by calculating pairwise Procrustes distances between each specimen for each shape partition and then computing matrix correlations between the pairwise distance matrices (Monteiro et al. 2005). The resulting correlation matrices were analyzed and visualized using graphical modeling (GM). The GM uses the assumption of conditional independence between the partitions of shape, and the hypotheses can be ranked using the deviance information criterion (DIC). Correlation matrices were produced using an Rscript developed by Adam Roundtree (available as supplementary material from Zelditch et al. 2012) and edited for its application with 3D landmark data and controlling for phylogeny (Larouche et al. 2022; available on Dryad). The GM was performed using the function *fitConGraph* from the R package *ggm* (Marchetti 2006).

The best-supported modularity hypothesis may differ between the CR method and the distance matrix approach. These methods use different metrics and test modularity in conceptually different ways. The covariance method uses the relative strengths of association in modules versus across modules and shows the pattern of integration across partitions of shape. Here, we defined the best-fitting hypothesis for the CR as the lowest effect size. The distance matrix approach takes conditional independence into consideration and emphasizes aspects of modularity that are quasi-independent (i.e., consequential for evolvability). There are two types of distance matrix tests, one that takes position and shape into consideration and one that only takes shape into account. We defined the best-fitting hypothesis as the one with the lowest DIC score. Together, these methods can help elucidate broad-scale modularity patterns by looking at the commonalities between the results.

### Evolutionary rate analyses

We used the rate ratio method (Denton and Adams 2015) to investigate whether the best-fitting modularity hypothesis for each tribe was paralleled by differences in phenotypic rates across modules. We ran these analyses on the best-fitting hypotheses from the two modularity analyses described above. The rate ratio method assumes a Brownian motion (BM) evolutionary model for each module, estimates per-module rates, and then calculates a ratio between the highest and lowest of these rates. These rate comparisons use the function *compare.evol.rates* for group-wise (i.e., across tribes) comparisons of the same landmark configurations and *compare.multi.evol.*rates for comparisons among subsets of landmarks belonging to these configurations, both implemented in the R package *geomorph* (Adams et al. 2022). We recognize that some of the modules may not evolve in BM; however, at this time, there is no method we can use to overcome this assumption. However, recent analyses suggest that the trends of the results are minimally influenced even if some partitions are not evolving under BM (Larouche et al. 2022).

### Linkage planarity metrics

To test the idea that linkage modularity may vary with the three dimensionality of linkage structure, we developed a metric of linkage planarity. Computational 4-bar linkage mechanics typically assumes that linkages are planar, with deviations from planarity resulting in some models being rejected as appropriate constructs for function, highlighting the need for 3D modeling (Westneat 1994; Olsen and Westneat 2016). The 3D coordinates of each 4-bar linkage were used to develop a metric of planarity to assess the degree to which the four rotational joints were positioned in the same plane in their rest positions. The fixed link (involving two joints) plus one mobile joint was aligned in the XY plane (with Z at zero), with the fourth joint retaining its Z-axis value, and the 3D area of the linkage was computed. Then, the fourth joint was projected onto the XY plane, and the projected planar area recalculated. Three-dimensional area was always greater than projected area, so the ratio of projected area to 3D area was used as a metric (ranging from 0 to 1.0) of the planarity of each of the three linkages across all 205 specimens.

## Results

The main results from our study are as follows: (1) the general pattern of modularity across the family Labridae is that the three linkage systems are highly integrated with each other but are separate modules from the neurocranium, nasals, premaxilla, and pharyngeal jaws; (2) linkage systems are conditionally independent in scarines and cheilines and evolve at faster diversification rates than in hypsigenyines and julidines; (3) the module containing all three linkage systems evolved faster than the remainder of the skull; and (4) the planarity of the linkages in their rest position is high in the anterior jaw linkage and opercular linkage but lower in the hyoid linkage.

### Phylomorphospace

We constructed multiple phylomorphospaces based on the shape data from the whole skull and for each individual tribe. For the whole skull phylomorphospace, PC1 explains the variation in rostral-caudal length (36.61% of the variation) of the skull. In addition, PC1 captures variation in urohyal length and positioning of the pectoral girdle (Fig. 3). PC2 explains the variation in dorsal-ventral length (15.26% of the variation) across the skull shapes. Additionally, PC2 captures variation in the supraoccipital crest and positioning of the pharyngeal jaws (Fig. 3). These two axes explain over half (51.87%) of the variation in wrasses.

Hypsigenyines’ first two axes of the phylomorphospace explain over half the variation in this tribe (67.18%). PC1 explains changes in rostral–caudal length as well as changes in the orbit, which account for 55.64% of the variation in this clade (Fig. 4). There is a strong stratified pattern along PC1 that is dominated by the species *Siphonognathus argyrophanes.* This species is an outlier among all wrasses and is characterized by an elongated skull with long premaxilla and dentary bones. PC2 captures 11.54% of the variation and describes changes in dorsoventral length and in urohyal length. Additionally, changes to the dorsal-ventral positioning of the hyoid apparatus, specifically the ceratohyal, are captured by PC2 (Fig. 4), indicating that PC2 captures the changes in the hyoid linkage system as well.

The julidine phylomorphospace shows PC1 containing 28.42% of shape change relating to the posterior region of the skull. More specifically, PC1 captures changes in shape of the operculum and pectoral girdle (Fig. 4). PC1 also captures the frontal–caudal length changes to the hyoid apparatus. PC1, therefore, captures changes to the opercular and hyoid linkage systems. On the other hand, PC2 captures 16.30% of variation and is dominated by variations in premaxilla, supraoccipital, and urohyal morphologies, indicating that PC2 captures some changes to the anterior jaw linkage system. Interestingly, there were no genus-related groupings in this tribe.

The cheiline phylomorphospace exhibits a PC1 and PC2 that capture a majority of variation in this tribe's skull (56.78%; Fig. 4). PC1 captures 39.28% variation, while PC2 captures 17.50%. The variation in PC1 is related to length changes in the lower jaw and urohyal, while PC2 captures variation in dorsal–ventral length. PC1 additionally captures rostral–caudal displacement of the nasals. Therefore, PC1 captures changes to the anterior jaw and hyoid linkage system, while PC2 captures changes in the opercular linkage system on the dorsal–ventral axis. *Oxycheilinus* mainly varies along PC1, while *Cheilinus* has most of its variation along PC2.

For the scarine phylomorphospace, shape change is captured by the primary axis of variation (PC1) that contains 26.46% of the overall variation and is dominated by changes in premaxilla angle and length and rostrocaudal length (Fig. 4). PC1 also mainly captures the placement of the lower pharyngeal jaw. In contrast, the next largest shape axis (PC2) contains 12.67% of the overall variation and explains differences in toothed versus beaked species. This indicates that both PC1 and PC2 capture changes to the anterior jaw linkage system. Additionally, PC2 captures dorsal–ventral variation, mainly with the hyoid linkage system. Several of the remaining axes of variation are related to the hyoid apparatus, pectoral girdle, and other aspects of the skull. About four PCs describe half the variation in scarines. We additionally see a stratified pattern along PC1 between the beaked and nonbeaked parrotfish (Fig. 4).

### Modularity tests

**Fig. 5:**
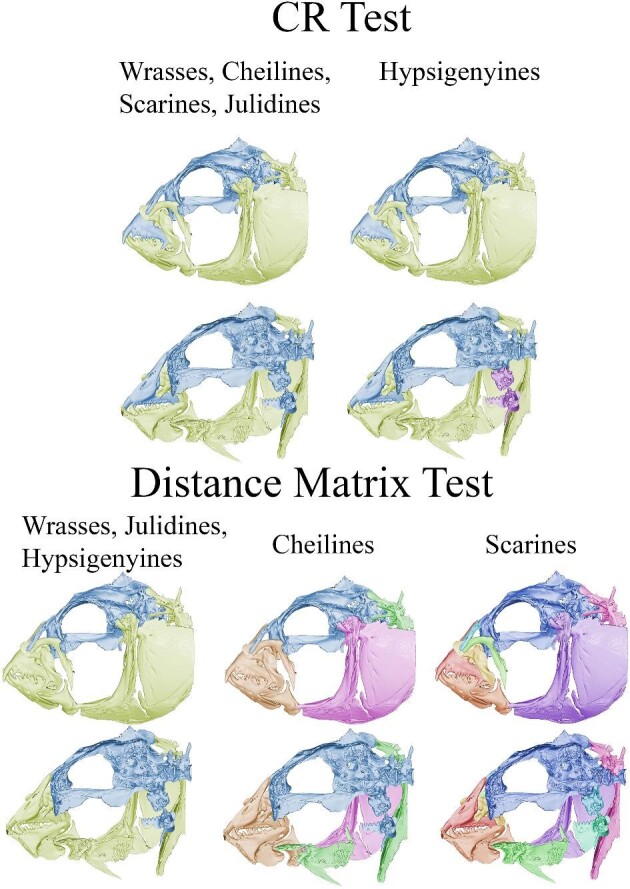
Modularity results represented on *Halichoeres argus* from the CR and distance matrix method modularity tests. Each color represents a different module where green is the three-linkage module and blue is the remainder of the bones (neurocranium, nasals, pharyngeal jaws, and premaxilla). In the CR test results, wrasses, cheilines, scarines, and julidines are being represented by hypothesis 3 while hypsigenyines represent hypothesis 10. In the distance matrix test, wrasses, julidines, and hypsigenyines represent hypothesis 2, cheilines represent hypothesis 8, and scarines represent hypothesis 1. All these hypotheses were best supported in the corresponding tests.

Modularity analysis across the entire family Labridae supports several of the hypotheses of modularity. The CR ratio test supports hypothesis 3, showing integration between all the linkages and placing the premaxilla in the remainder of the skull module (i.e., nasals, neurocranium, premaxilla, and pharyngeal jaws; Supplementary Table 4). The GM supports hypothesis 2 in both tests (Supplementary Table 5), which indicates that the premaxilla is integrated with the linkages. The support for these hypotheses indicates integration of the three linkages with one another, which is the main pattern of modularity throughout wrasses. Furthermore, these linkages are decoupled from the remainder of the skull, including the upper and lower pharyngeal jaws (Fig. 5).

Analysis of the hypsigenyines supported three hypotheses of modularity (Table 2). The CR test supports hypothesis 10 (Supplementary Table 6), a three-module hypothesis with all the linkages integrated into one module, with the nasals and neurocranium making a second and the pharyngeal jaws making a third module. The GM supported hypotheses 3 and 5 (Supplementary Table 7). Hypothesis 3 is a two-module hypothesis and partitions the data into the linkage bones (e.g., the bones that compose the 4-bar linkage systems) and the remainder of the skull. This hypothesis is supported by the graphical modeling test that preserves relative positions and sizes in the data. Hypothesis 5 is a four-module hypothesis in which the three linkages compose three modules with the premaxilla, and the remainder of the bones are in the fourth module. This hypothesis is supported by the GM test, which only considers variation in shapes of landmark positions. We reran our analyses without the outlier *S. argyrophanes* and found similar results in that the hypotheses supported grouped the linkages together as one module. These hypotheses indicate that in hypsigenyines, the linkages are integrated in all variables except shape, where the linkages are more modular. This indicates that the bones involved with each linkage are able to vary in shape but are linked to each other in position and size.

**Table 2:** Modularity hypothesis support for the covariance ratio (CR) and graphical modeling (GM) methods

Tribe	Test	Hypothesis supported	*P*-value	effect size	DIC score
Hypsigenyines	CR	10	0.001	−10.54	N/a
	GM	3	N/a	N/a	−37.652346
	GM	5	N/a	N/a	−76.026436
Julidines	CR	2	0.001	−20.000761	N/a
	GM	2	N/a	N/a	2.45716529
	GM	4	N/a	N/a	−19.694909
Cheilines	CR	2	0.001	−17.36	N/a
	GM	8	N/a	N/a	−80.949597
	GM	9	N/a	N/a	−126.36137
Scarines	CR	3	0.001	−10.52	N/a
	GM	12	N/a	N/a	−96.073491
	GM	1	N/a	N/a	−183.58255

The julidine data support two modularity hypotheses. The CR test supports hypothesis 2 (Supplementary Table 8), while graphical modeling supports hypotheses 2 and 4 (Table 2; Supplementary Table 9). Hypothesis 2 has two distinct modules with the premaxilla being treated as a part of the linkage systems. Hypothesis 4 has four distinct modules and splits the linkage systems into three with the remainder of the bones, including the premaxilla, as the fourth module. The GM method that emphasizes shape variation supports hypothesis 4 (Supplementary Table 9), suggesting strong covariation in shape *in* linkage systems, whereas covariation in position and size is predominant *between* the linkage systems. Julidines have a similar pattern to the hypsigenyines in that the bones involved in linkages are able to vary in shape more independently but are linked in all other variables.

Cheilines support three hypotheses of modularity: hypotheses 2, 8, and 9 (Table 2). The CR test supports hypothesis 2 (Supplementary Table 10), while GM supports hypotheses 8 and 9 (Supplementary Table 11). Hypothesis 2 is a two-module hypothesis with the premaxilla being treated as a part of the linkage module. Hypotheses 8 and 9 both demarcate the linkage systems as their own modules. These two hypotheses indicate that the linkages are conditionally independent when position and shape are considered. However, there is still strong covariation across the linkages compared to the bones not involved in the linkages based on the CR test supporting hypothesis 2. We reran our analyses without the outlier, *Epibulus insidiator*, and found similar results that split the linkages into three different modules. The support for these three hypotheses indicates that cheilines deviate from the pattern of modularity at the family level. Cheilines’ linkages have become more modular and more independent from each other.

Finally, the scarine data support three modularity hypotheses (Table 2). The CR test supports hypothesis 3 (Supplementary Table 12). This hypothesis has the three linkages covarying but independent from the remainder of the skull with the premaxilla. The graphical modeling method supports hypotheses 1 and 12 (Supplementary Table 13). Hypothesis 1 separates each bone into its own module, whereas in hypothesis 12 there are five modules with each linkage system being a separate module, alongside a fourth module including the neurocranium and nasals and a fifth module including the pharyngeal jaws. This indicates that there may be increased modularization across scarines due to the majority of the tests supporting hypotheses that treat the linkages as separate modules and the remainder of the skull parsing into two more additional modules.

### Evolutionary rates of change in skull shape

We calculated the net rate of shape evolution for the whole skull and across all labrid tribes. Additionally, we computed the rate of shape evolution for each 4-bar linkage system across Labridae, represented as the bones connected in the linkage system. The cheilines have the highest rate of evolution for every shape tested (Table 3). We additionally calculated the shape change for the bones associated with the linkages. We found a trend in which the hyoid module is the fastest evolving system, followed by the anterior jaw module, opercular module, and neurocranium module (Table 4). Additionally, the linkages having a higher rate of evolution than the remainder of the bones held true when rates were estimated and compared between a linkage module (i.e., all linkages are included in a single module) and a second module comprising the remainder of the bones (Table 4).

**Table 3:** Comparison of the whole skull and three linkage systems across the four tribes analyzed

Tribe	Structure	Evolutionary rate
Cheilines	Whole skull	9.85E−05
Hypsigenyines	Whole skull	2.69E−06
Julidines	Whole skull	3.32E−06
Scarines	Whole skull	4.13E−06
Cheilines	Anterior linkage	6.45E−05
Hypsigenyines	Anterior linkage	2.69E−06
Julidines	Anterior linkage	2.44E−06
Scarines	Anterior linkage	3.03E−06
Cheilines	Opercular linkage	6.47E−05
Hypsigenyines	Opercular linkage	1.88E−06
Julidines	Opercular linkage	2.09E−06
Scarines	Opercular linkage	2.54E−06
Cheilines	Hyoid linkage	3.31E−04
Hypsigenyines	Hyoid linkage	4.25E−06
Julidines	Hyoid linkage	7.41E−06
Scarines	Hyoid linkage	7.94E−06

**Table 4:** Evolutionary rates for shape across all 205 species. The remainder of the skull includes the neurocranium, nasals, premaxilla, and pharyngeal jaws

Structure	Evolutionary rate
Anterior jaw linkage	1.12E−05
Opercular linkage	9.70E−06
Hyoid linkage	4.06E−05
Remainder of the skull	8.87E−06
Linkage module	2.01E−05

We analyzed the four tribes independently and found that the hypsigenyine and julidine patterns of modularity support integration between linkages. Overall, the hypsigenyine and julidine trends are the evolutionary rates of the linkage module evolve at a faster rate than the neurocranium (Table 3).

Cheilines show a pattern of modularity in which the three linkage systems are independent modules. The net change of shape for the bones associated with the anterior linkage system was the lowest, followed by the opercular and hyoid linkage systems. The upper pharyngeal jaw has a diversification rate that is higher than that of the lower pharyngeal jaws (Supplementary Table 14). Scarines show a pattern of modularity that indicates each bone is its own module. The net shape diversification rates of each bone from lowest to highest were nasals, hyomandibula, premaxilla, dentary, maxilla, neurocranium, articular, operculum, palatine, pectoral girdle, upper pharyngeal jaws, lower pharyngeal, ceratohyal, and urohyal. Overall, in the intra-tribe analyses, the bones associated with the linkages are mostly evolving at faster rates compared to the remainder of the bones in the skull.

### Linkage planarity

The anterior jaw linkage and opercular linkage were highly planar in their rest positions, with a mean planarity value of about 0.9 for both systems, and often ranging up to 1.0, indicating that the four linkage joints occupy the same plane (Supplementary Table 15). The maximum planarity for the anterior jaw linkage system was 1.0 in *Pseudolabrus guentheri* with the minimum in *Ctenolabrus rupestris* at 0.62. Similarly, the planarity for the opercular linkage system ranged from a maximum of 1.0 in *Chlorurus microrhinos* down to 0.41 in *E. insidiator.* In contrast, the most rapidly evolving linkage, the hyoid linkage, was typically less planar, with a mean planarity of 0.72, ranging from 0.96 in *Labropsis australis* to 0.52 in *Leptoscarus vaigiensis.*

## Discussion

An important question at the interface of phylogenetics and biomechanics is the degree to which important musculoskeletal systems, such as the forelimbs in tetrapods or the skulls of teleost fishes, coevolve in an integrated way or evolve relatively independent of one another. Here, we reveal a strong pattern of modularity across the reef fish family Labridae in which the 4-bar linkage systems are often integrated as a unit, vary across groups in whether they coevolve with each other, and evolve independently from the remainder of the skull. Hypsigenyines and julidines generally supported a model of integration across the linkage systems, similar to the family-wide patterns. Cheilines are an anatomical and functional hotspot of linkage evolution, as the three linkage systems are mostly all separate modules, and they have the fastest rates of linkage evolution in the family. The parrotfishes (scarine wrasses) are also extreme, showing a pattern of increased modularization of the skull. Among the linkages, the hyoid is the fastest evolving linkage system, and the least planar in its rest position, with the anterior jaws and opercular system showing somewhat lower evolutionary rates of change in shape and a mostly planar geometry. We conclude that the biomechanical systems in the labrid skull affect the modularity and integration of the bones in the skull and that the trade-off of integration versus modularity of biomechanical systems influences the tempo of skull evolutionary shape change in the labrid fishes.

### Labrid phylomorphospace and the modularity of 4-bar linkage systems

The central conclusion of this study is that biomechanical 4-bar linkages in the skull of labrid fishes show a strong pattern of evolutionary modularity across the family, with labrid subclades showing different levels of integration and evolutionary rates among linkage modules. Recent work exploring this 3D morphometric data set on the skull of labrid fish has highlighted the independent modular evolution of the neurocranium and pharyngeal jaws (Evans et al. 2019), revealed the morphometric constraints in the skull shape related to burrowing behavior (Evans et al. 2022), and discovered significant functional modularity and mosaic patterns of evolution in the labrid skull (Larouche et al. 2022). Here, we extend the analysis of these data to specifically test evolutionary modularity in the 4-bar linkage mechanisms that function in the feeding apparatuses of this diverse group.

Hypsigenyines and julidines have similar linkage modularity patterns to each other and the family Labridae. Most tests in hypsigenyines support a single linkage module with the remainder of the skull being one or two modules. The support for a three-module hypothesis may indicate a small release of integration in this tribe. This may explain why there is widespread in the phylomorphospace (Fig. 4) reflecting a high diversity in feeding ecologies and deep-water forms in this tribe. The release in constraint may have also allowed the extreme variation in *S. argyrophanes* to evolve as an extreme morphology in the phylomorphospace of wrasses (Fig. 3).

Similarly, in julidines, the linkage systems form a single module, and this is supported in the majority of the modularity tests conducted. This pattern may indicate some constraint on the julidines’ skull, although this tribe is a catch-all assemblage of many genera forming the diverse crown of the phylogeny (Hughes et al. 2022), some of which are paraphyletic, which may confound the results. The skull of julidines appears to have radiated in shape to fill most of the phylomorphospace (Fig. 4), and there are many convergences between julidine taxa and other genera among wrasses in the phylomorphospace (Fig. 3). The integration of the three linkage systems may have spurred the evolution of specialty feeding behaviors (i.e., cleaners) not found in the other tribes and may have driven patterns of convergent morphology in the phylomorphospace. Further investigations into individual species patterns of modularity should be conducted to determine the effect of constraint on the morphology of the skull. Additional investigations into the phylomorphospace of julidine subgroups with increased sampling, such as the multiple radiations of *Halichoeres*, might further reveal constraint on the morphology of the julidine skull.

Two hotspots of skull and linkage evolution in the family Labridae identified here are the tribes Cheilini and Scarini, the labrid groups with the majority of hypotheses supporting the most independent linkage modules, the least integrated skull components, and the highest rates of shape change (Table 3). The independence of the linkage systems may have enabled morphological innovations to evolve in these two groups.

Cheiline wrasses are morphologically and ecologically diverse, including piscivores and hard-shelled molluscivores, with size ranging from the smallest wrasse *Wetmorella nigropinnata* to the largest *Cheilinus undulatus* (Westneat 1993). Research on the cheiline wrasse genera (*Cheilinus, Epibulus*, and *Oxycheilinus*) reveals the actions of planar 4-bar linkage models, which have been directly tested and supported with live animal feeding kinematics (Westneat 1990, 1991, 1994). In cheilines, morphological innovations have evolved in the 4-bar linkage systems with linkage modifications for biting and piscivory. This includes the highly modified 6-bar anterior jaw linkage system in the sling-jaw wrasses, *Epibulus* (Westneat 1991). The support for increased modularization of the three linkage systems may have allowed for more independent evolution between the linkage systems in cheilines associated with their ecomorphological diversification, restructured functional traits, and elevated levels of linkage modularity and rates of shape change. It is interesting that the quasi-independence of the three linkage systems gave way to a general pattern of mobility in cheilines, which may point to cranial kinesis’ ability to reorganize and restructure functional traits and can result in differences in modularity and rates of diversification.

In scarines, a model of increased modularization is mainly supported compared to the hypsigenyines and julidines. The Scarini is composed of the parrotfish, which vary in jaw morphology from the partially fused teeth of *Sparisoma* to the fully fused beak of the genus *Scarus*. In addition to changes in tooth morphology, some scarines also exhibit a mobile intramandibular joint in their lower jaw (i.e., anterior jaw linkage system; Price et al. 2010). Previous research on parrotfish has shown moderate diversity in their skull shape (Wainwright et al. 2004) and high partitioning in their feeding habits (Nicholson and Clements 2020), leading these fish to occupy a largely separate region of the labrid phylomorphospace (Fig. 3) This divergence of the scarines has been previously found in analyses of linear measures and muscle metrics (Wainwright et al. 2004) as well as 3D geometric morphometrics of the labrid skull (Larouche et al. 2022). Additionally, parrotfish have elevated diversification rates, with most *Scarus* and *Chlorurus* species diversifying in just the past 5–10 million years (Smith et al. 2008; Hughes et al. 2022) with elevated rates of morphological evolution associated with rapid diversification (Price et al. 2010), which may further drive the diversity in these fish.

Overall family trends show more independence of the linkages, which allows for more variability in the linkage systems, providing more evidence that modularity can lead to innovative morphologies (Wagner and Altenberg 1996; Tokita et al. 2007; Hansen and Houle 2008; Clune et al. 2013). New morphologies in the anterior 4-bar linkage system evolved in the cheilines and scarines, which show patterns of increased modularization, which indicates a tight relationship between the 4-bar linkage systems and the morphology and shape of the skull in wrasses. It is intriguing that hypsigenyines and julidines show similar modularity patterns, even though they are distantly related to one another. Based off phylogenetic positioning, the ancestral state of modularity patterns appears to be an integrated skull with the linkages and the remainder of the skull as separate modules. Future studies should investigate the other tribes (labrines, cirrhilabrines, pseudolabrines, and novaculines) to understand if there is convergent evolution in modularity patterns of the skull. Furthermore, this points to function and the corresponding mechanical systems potentially being more of a proximal driver of evolutionary modularity.

### Modularity related to evolutionary rates of shape

Four-bar linkage systems in the skull of labrid fishes show elevated rates of evolutionary shape diversification relative to the rest of the skull (neurocranium, nasals, pharyngeal jaws, and premaxilla; Table 3). We conclude that elevated rates of linkage change are an expression of the diversity of feeding mechanisms enabling the global ecological diversification of this iconic reef fish family. Several recent studies have explored the relationship between integration and rates of diversification, quantifying phenotypic and evolutionary modularity to determine how they relate to these variables in various species and clades and providing evidence for a relationship in which more modular structures have either higher or lower evolutionary rates (Goswami and Polly 2010; Claverie and Patek 2013; Larouche et al. 2018; Bardua et al. 2019; Evans et al. 2019). However, some studies have found no relationship between these variables (Bardua et al. 2019, 2020; Bon et al. 2020), indicating a relationship that is complex. In our study, we found an apparent association between modularity and the evolutionary rates of skull shape across the labrid phylogeny, with the species showing increased modularization having higher rates of shape evolution (Table 3) and the linkage system module evolving at a faster rate of shape diversification than the neurocranium, nasals, premaxilla, and pharyngeal jaws (Table 3).

The concept of evolvability has been used to assess the potential for evolutionary change and has been used to examine anatomical modularity and evolutionary rates of change (Wagner et al. 2007; Pigliucci 2008; Clune et al. 2013). The 4-bar linkage systems evolve at about double the rate of the neurocranium, nasals, premaxilla, and pharyngeal jaws (Table 4). These differences in evolutionary shape change among skull modules signify elevated evolvability in these systems, likely related to the strong pattern of ecomorphological diversification of labrid fish (Wainwright et al. 2004) that is accompanied by rapid divergence in feeding biomechanics among closely related lineages (Westneat et al. 2005). The linkage modules directly influence the performance and success of prey capture, which may be driving the evolvability of these regions. The evolvability of the linkage systems may have allowed for new morphologies to evolve and thus tribes, such as scarines and cheilines, moved into new niches and habitats. However, having more independence in a structure can confer only so much of a benefit to the species. If it is also accompanied by rate differences in the modules, further influence of modularity could affect the evolution of a structure. The spectacular ecomorphological diversity of the labrid fish across reef systems is associated with different levels of modularity in the skull and elevated rates of shape change in the configuration of the complex skull levers and linkages involved in feeding mechanisms.

#### Planarity versus three dimensionality of linkage systems

An important finding of our 3D linkage analysis is that the anterior jaws and opercular linkages are highly planar, with all four rotational joints aligned close to a plane. A key conclusion from this result is that the most 3D linkage system, the hyoid, is the fastest evolving in shape (Table 3). Additionally, kinematic analyses and computational modeling studies that start from an assumption of planar linkage positioning are supported, at least for the initial starting position. The 4-bar linkage systems have been modeled as 2D structures (Westneat 1990) and as 3D structures (Olsen et al. 2017). Moving forward, researchers need to especially treat the hyoid linkage system as a 3D structure. This allows more precise measurements to be taken from this system. This also leads to a question of why the hyoid linkage system is so three dimensional compared to the other systems? And why is this system the fastest evolving?

The three dimensionality of the hyoid linkage system is clearly related to complex 3D hyoid kinematics in many fish groups (Van Wassenbergh et al. 2007; Camp and Brainerd 2014, 2015). The main motions of the hyoid linkage system are depression and retraction. The retraction drives some lower jaw depression, while the depression influences the lateral expansion of the skull during feeding and hyoid depression. A more 3D linkage system would allow greater lateral and ventral expansion of the skull (Olsen et al. 2017; Gartner et al. 2022; Whitlow et al. 2022). Furthermore, changes to this system occur faster due to the high rate of shape change in the hyoid linkage system. This lateral movement may be why the hyoid system is so three-dimensional compared to the anterior jaw and opercular linkage systems. More lateral movement would increase the oral cavity volume and would increase the pressure differential created during suction feeding (Lauder 1983). This would also benefit biting species as it would draw the prey further into the oral cavity, bringing the food to the pharyngeal jaws to be broken down.

## Conclusion

In summary, we find the shapes of the bones associated with the linkage systems to be evolving more independently and faster than the remainder of the skull shape (i.e., neurocranium, nasals, pharyngeal jaws, and premaxilla) in the family Labridae. An analysis of family-wide and tribe-wide modularity patterns shows evolution of different modularity patterns across the tree where the scarines and cheilines show evolution of increased modularization of the skull and faster rates of shape evolution, relative to other clades in the family. We conclude that variable modularity and elevated rates of shape evolution contribute to the morphological and functional novelties in the anterior jaw linkage system and drive patterns of shape evolution in wrasses. The planar positions of the jaws and opercular linkages, and the high level of three-dimensionality of the hyoid linkage system, will also help to inform future computational analyses on these linkage systems in wrasses.

## Supplementary Material

obad035_Supplemental_Files

## References

[bib1] Adams DC, Collyer ML. 2019. Comparing the strength of modular signal, and evaluating alternative modular hypotheses, using covariance ratio effect sizes with morphometric data. Evolution (N Y) 73:2352–67. 10.1111/evo.13867 31657008

[bib2] Adams DC, Collyer ML, Kaliontzopoulou A, Baken EK. 2022. Geomorph: software for geometric morphometric analyses. R package version 4.0.4.

[bib3] Aiello BR, Westneat MW, Hale ME. 2017. Mechanosensation is evolutionarily tuned to locomotor mechanics. Proc Natl Acad Sci 114:4459–64. 28396411 10.1073/pnas.1616839114 PMC5410822

[bib4] Albertson RC, Streelman JT, Kocher TD, Yelick PC. 2005. Integration and evolution of the cichlid mandible: the molecular basis of alternate feeding strategies. Proc Natl Acad Sci U S A 102:16287–92. 16251275 10.1073/pnas.0506649102 PMC1283439

[bib5] Anker GC . 1974. Morphology and kinetics of the head of the stickleback, *Gasterosteus aculeatus*. Trans Zool Soc London 32:311–416.

[bib6] Bardua C, Fabre AC, Bon M, Das K, Stanley EL, Blackburn DC, Goswami A. 2020. Evolutionary integration of the frog cranium. Evolution (N Y) 74:1200–15. 10.1111/evo.13984 32346857

[bib7] Bardua C, Wilkinson M, Gower DJ, Sherratt E, Goswami A. 2019. Morphological evolution and modularity of the caecilian skull. BMC Evol Biol 19:1–23. 30669965 10.1186/s12862-018-1342-7 PMC6343317

[bib8] Barel CDN . 1982. Towards a constructional morphology of cichlid fishes (Teleostei, Perciformes). Netherlands J Zool 357–424.

[bib9] Baumgart A, Anderson P. 2018. Finding the weakest link: mechanical sensitivity in a fish cranial linkage system. R Soc Open Sci 5:181003. 30473846 10.1098/rsos.181003 PMC6227944

[bib10] Bon M, Bardua C, Goswami A, Fabre AC. 2020. Cranial integration in the fire salamander, *Salamandra salamandra* (Caudata: salamandridae). Biol J Linn Soc 130:178–94.

[bib11] Camp AL, Brainerd EL. 2014. Role of axial muscles in powering mouth expansion during suction feeding in largemouth bass *(Micropterus salmoides)*. J Exp Biol 217:1333–45. 24363416 10.1242/jeb.095810

[bib12] Camp AL, Brainerd EL. 2015. Reevaluating musculoskeletal linkages in suction-feeding fishes with X-ray reconstruction of moving morphology (XROMM). Integr Comp Biol 55:36–47. 25972567 10.1093/icb/icv034

[bib13] Cardini A, O'Higgins P, Rohlf FJ. 2019. Seeing distinct groups where there are none: spurious patterns from between-group PCA. Evol Biol 46:303–16.

[bib14] Cheverud JM . 1982. Phenotypic, genetic, and environmental morphological integration in the cranium. Evolution (N Y) 36:499. 10.1111/j.1558-5646.1982.tb05070.x 28568050

[bib15] Cheverud JM . 1995. Morphological integration in the saddle-back tamarin *(Saguinus fuscicollis)* cranium. Am Nat 145:63–89.

[bib16] Churchill M, Miguel J, Beatty BL, Goswami A, Geisler JH. 2019. Asymmetry drives modularity of the skull in the common dolphin *(Delphinus delphis)*. Biol J Linn Soc 126:225–39.

[bib17] Claverie T, Patek SN. 2013. Modularity and rates of evolutionary change in a power-amplified prey capture system. Evolution (N Y) 67:3191–207. 10.1111/evo.12185 24152002

[bib18] Clune J, Mouret JB, Lipson H. 2013. The evolutionary origins of modularity. Proc R Soc B Biol Sci 280:20122863. 10.1098/rspb.2012.2863 PMC3574393 23363632

[bib19] Denton JSS, Adams DC. 2015. A new phylogenetic test for comparing multiple high-dimensional evolutionary rates suggests interplay of evolutionary rates and modularity in lanternfishes (Myctophiformes; Myctophidae). Evolution (N Y) 69:2425–40. 10.1111/evo.12743 26278586

[bib20] Evans KM, Larouche O, West JJL, Gartner SM, Westneat MW. 2022. Burrowing constrains patterns of skull shape evolution in wrasses. Evol Dev 73–84. 35971630 10.1111/ede.12415

[bib21] Evans KM, Vidal-García M, Tagliacollo VA, Taylor SJ, Fenolio DB. 2019. Bony patchwork: mosaic patterns of evolution in the skull of electric fishes (Apteronotidae: gymnotiformes). Integr Comp Biol 59:420–31. 31070738 10.1093/icb/icz026

[bib22] Evans KM, Waltz B, Tagliacollo V, Chakrabarty P, Albert JS. 2017. Why the short face? Developmental disintegration of the neurocranium drives convergent evolution in neotropical electric fishes. Ecol Evol 7:1783–1801. 28331588 10.1002/ece3.2704 PMC5355199

[bib23] Gartner SM, Whitlow KR, Laurence-Chasen JD, Kaczmarek EB, Granatosky MC, Ross CF, Westneat MW. 2022. Suction feeding of West African lungfish *(Protopterus annectens)*: an XROMM analysis of jaw mechanics, cranial kinesis, and hyoid mobility. Biol Open 11:bio059447. 36066131 10.1242/bio.059447 PMC9493713

[bib24] Goswami A, Polly PD. 2010. Methods for studying morphological integration and modularity. Paleontol Soc Pap 16:213–43.

[bib25] Goswami A, Watanabe A, Felice RN, Bardua C, Fabre AC, Polly PD. 2019. High-density morphometric analysis of shape and integration: the good, the bad, and the not-really-a-problem. Integr Comp Biol 59:669–83. 31243431 10.1093/icb/icz120 PMC6754122

[bib26] Hansen TF, Houle D. 2008. Measuring and comparing evolvability and constraint in multivariate characters. J Evol Biol 21:1201–19. 18662244 10.1111/j.1420-9101.2008.01573.x

[bib27] Hughes LC, Nash CM, White WT, Westneat MW. 2022. Concordance and discordance in the phylogenomics of the wrasses and parrotfishes (Teleostei: labridae). Syst Biol 72:530–43. 10.1093/sysbio/syac072 36331534

[bib28] Klingenberg CP . 2008. Morphological integration and developmental modularity. Annu Rev Ecol Evol Syst 39:115–32.

[bib29] Larouche O, Gartner SM, Westneat MW, Evans KM. 2022. Mosaic evolution of the skull in labrid fishes involves differences in both tempo and mode of morphological change. Syst Biol 72:419–32. 10.1093/sysbio/syac061 36111797

[bib30] Larouche O, Zelditch ML, Cloutier R. 2018. Modularity promotes morphological divergence in ray-finned fishes. Sci Rep 8:1–6. 29740131 10.1038/s41598-018-25715-y PMC5940925

[bib31] Lauder BYGV . 1983. Prey capture hydrodynamics in fishes: experimental tests of two models. J Exp Biol 104:1–13.

[bib32] Lauder GV . 1982. Patterns of evolution in the feeding mechanism of actinopterygian fishes. Am Zool 22:275–85.

[bib33] Liem KF . 1970. Comparative functional anatomy of the nandidae (Pisces: teleostei). Fieldiana Zool 56:1–166.

[bib34] Marchetti GM . 2006. Independencies induced from a graphical markov model after marginalization and conditioning: the R package ggm. J Stat Softw 15:1–15.

[bib35] Monteiro LR, Bonato V, Dos Reis SF. 2005. Evolutionary integration and morphological diversification in complex morphological structures: mandible shape divergence in spiny rats (Rodentia, Echimyidae). Evol Dev 7:429–39. 16174036 10.1111/j.1525-142X.2005.05047.x

[bib36] Muller M . 1989. A quantitative theory of expected volume changes of the mouth during feeding in teleost fishes. J Zool London 217:639–62.

[bib37] Nicholson GM, Clements KD. 2020. Resolving resource partitioning in parrotfishes (Scarini) using microhistology of feeding substrata. Coral Reefs 39:1313–27.

[bib38] Olsen AM, Camp AL, Brainerd EL. 2017. The opercular mouth-opening mechanism of largemouth bass functions as a 3D four-bar linkage with three degrees of freedom. J Exp Biol 220:4612–23. 29237766 10.1242/jeb.159079

[bib39] Olsen AM, Westneat MW. 2016. Linkage mechanisms in the vertebrate skull: structure and function of three-dimensional, parallel transmission systems. J Morphol 277:1570–83. 27577864 10.1002/jmor.20596

[bib40] Olson EC, Miller RL. 1999. Morphological integration. University of Chicago Press.

[bib41] Ornelas-García CP, Bautista A, Herder F, Doadrio I. 2017. Functional modularity in lake-dwelling characin fishes of Mexico. PeerJ 2017:1–22. 10.7717/peerj.3851 PMC5611896 28951817

[bib42] Paradis E, Claude J, Strimmer K. 2004. APE: analyses of phylogenetics and evolution in R language. Bioinformatics 20:289–90. 14734327 10.1093/bioinformatics/btg412

[bib43] Parsons KJ, Cooper WJ, Albertson RC. 2011. Modularity of the oral jaws is linked to repeated changes in the craniofacial shape of African cichlids. Int J Evol Biol 2011:1–10. 10.4061/2011/641501 PMC3119590 21716745

[bib44] Parsons KJ, Márquez E, Craig Albertson R. 2012. Constraint and opportunity: the genetic basis and evolution of modularity in the cichlid mandible. Am Nat 179:64–78. 22173461 10.1086/663200

[bib45] Pigliucci M . 2008. Is evolvability evolvable? Nat Perspect 9:75–82. 10.1038/nrg2278 18059367

[bib46] Price SA, Wainwright PC, Bellwood DR, Kazancioglu E, Collar DC, Near TJ. 2010. Functional innovations and morphological diversification in parrotfish. Evolution (N Y) 64:3057–68. 10.1111/j.1558-5646.2010.01036.x 20497217

[bib47] Revell LJ . 2012. phytools: an R package for phylogenetic comparative biology (and other things). Methods Ecol Evol 3:217–23.

[bib48] Rohlf FJ, Slice D. 1990. Extensions of the procrustes method for the optimal superimposition of landmarks. Syst Zool. 39:40–59.

[bib49] RStudio . 2012. RStudio: integrated development environment for R. J Wildl Manage 770:165–71.

[bib50] Sidlauskas B . 2008. Continuous and arrested morphological diversification in sister clades of characiform fishes: a phylomorphospace approach. Evolution (N Y) 62:3135–56. 10.1111/j.1558-5646.2008.00519.x 18786183

[bib51] Smith LL, Fessler JL, Alfaro ME, Streelman JT, Westneat MW. 2008. Phylogenetic relationships and the evolution of regulatory gene sequences in the parrotfishes. Mol Phylogenet Evol 49:136–52. 18621133 10.1016/j.ympev.2008.06.008 PMC3418665

[bib52] Tokita M, Kiyoshi T, Armstrong KN. 2007. Evolution of craniofacial novelty in parrots through developmental modularity and heterochrony. Evol Dev 9:590–601. 17976055 10.1111/j.1525-142X.2007.00199.x

[bib53] Van Wassenbergh S, Herrel A, Adriaens D, Aerts P. 2007. Interspecific variation in sternohyoideus muscle morphology in clariid catfishes: functional implications for suction feeding. J Morphol 268:232–42. 17265443 10.1002/jmor.10510

[bib54] Wagner GP . 1996. Homologues, natural kinds and the evolution of modularity. Am Zool 36:36–43.

[bib55] Wagner GP, Altenberg L. 1996. Complex adaptations and the evolution of evolvability. Evolution (N Y) 50:967–76. 10.1111/j.1558-5646.1996.tb02339.x 28565291

[bib56] Wagner GP, Pavlicev M, Cheverud JM. 2007. The road to modularity. Nat Rev Genet 8:921–31. 18007649 10.1038/nrg2267

[bib57] Wainwright PC, Bellwood DR, Westneat MW, Grubich JR, Hoey AS. 2004. A functional morphospace for the skull of labrid fishes: patterns of diversity in a complex biomechanical system. Biol J Linn Soc 82:1–25.

[bib58] Westneat MW . 1990. Feeding mechanics of teleost fishes (Labridae; Perciformes): a test of four-bar linkage models. J Morphol 205:269–95. 29865760 10.1002/jmor.1052050304

[bib59] Westneat MW . 1991. Linkage biomechanics and evolution of the unique feeding mechanism of *Epibulus insidiator* (Labridae: teleostei). J Exp Biol 159:165–84.

[bib60] Westneat MW . 1993. Phylogenetic relationships of the tribe cheilinini (Labridae: perciformes). Bull Mar Sci 52:351–94.

[bib61] Westneat MW . 1994. Transmission of force and velocity in the feeding mechanisms of labrid fishes (Teleostei, Perciformes). Zoomorphology 114:103–18.

[bib62] Westneat MW . 2006. Skull biomechanics and suction feeding in fishes. Fish Physiol 23:29–75.

[bib63] Westneat MW, Alfaro ME. 2005. Phylogenetic relationships and evolutionary history of the reef fish family labridae. Mol Phylogenet Evol 36:370–90. 15955516 10.1016/j.ympev.2005.02.001

[bib64] Westneat MW, Alfaro ME, Wainwright PC, Bellwood DR, Grubich JR, Fessler JL, Clements KD, Smith LL. 2005. Local phylogenetic divergence and global evolutionary convergence of skull function in reef fishes of the family labridae. Proc R Soc B. Biol Sci. 272:993–1000. 10.1098/rspb.2004.3013 PMC1599880 16024356

[bib65] Whitlow KR, Ross CF, Gidmark NJ, Laurence-Chasen JD, Westneat MW. 2022. Suction feeding biomechanics of *Polypterus bichir*: investigating linkage mechanisms and the contributions of cranial kinesis to oral cavity volume change. J Exp Biol 225:jeb243283. 35019979 10.1242/jeb.243283

[bib66] Zelditch ML, Swiderski DL, Sheets HD. 2012. Geometric morphometrics for biologists: a primer. USA: Academic Press.

