# Correction to: Evolutionary Patterns of Modularity in the Linkage Systems of the Skull in Wrasses and Parrotfish

**DOI:** 10.1093/iob/obad040

**Published:** 2023-12-15

This is a correction to: S. M. Gartner, O. Larouche, K. M. Evans, M. W. Westneat, Evolutionary Patterns of Modularity in the Linkage Systems of the Skull in Wrasses and Parrotfish, *Integrative Organismal Biology*, Volume 5, Issue 1, 2023, obad035, https://doi.org/10.1093/iob/obad035

In the originally published version of this manuscript, the terms “parrotfishes” and other instances of the plural form “fishes” were given as “parrotfish” and “fish”, an incorrect plural form for multiple species. Therefore the plural form fish has been changed to fishes at several points throughout the manuscript, including the title.

“Fig. 2 Time-calibrated tree of the Labridae (410 species) from Larouche et al. 2022 with red bars indicating presence of the 205 wrasse species in our data set while **colored branches** point to the tribes we analyzed further in the modularity study: (A) hypsigenyines highlighted in orange, (B) julidines highlighted in blue, (C) cheilines highlighted in green, and (D) scarines highlighted in pink. A scale bar is provided on the bottom right in million years per before present (mybp).”

This error has been corrected online.

