# Supplementary material for: Evolutionary Patterns of Modularity in the Linkage Systems of the Skull in Wrasses and Parrotfishes
Source: Integr Org Biol. 2023 Sep 26;5(1):obad035. doi: 10.1093/iob/obad035 (PMC10583192; doi:10.1093/iob/obad035)
Supplement: obad035_Supplemental_Files [file obad035_supplemental_files.zip › SuppTable 2-3.docx]

**Table S2:** Adapted from Evans et al. 2022. Materials examined for the three-dimensional geometric morphometric analysis of 206 skull shape in wrasses. BMNH= British Museum of Natural History, ANSP= Academy of Natural Sciences of Drexel University, FMNH= Field Museum of Natural History, uncat= uncatalogued, AMNH= American museum of Natural History, AM= Australian Museum, UWFC= Burke Museum of Natural History, USNM=Smithsonian Museum of Natural history, BPBM= Bernice Pauahi Bishop Museum.

| **Species** | **Museum Code** | **Catalog number** | |
| --- | --- | --- | --- |
| *Acantholabrus palloni* | BMNH | | 5492 |
| *Achoerodus gouldii* | ANSP | | 13450 |
| *Anampses chrysocephalus* | FMNH | | 63668 |
| *Anampses cuvier* | FMNH | | 63669 |
| *Anampses femininus* | Uncat | |  |
| *Anampses geographicus* | FMNH | | 110638 |
| *Anampses melanurus* | Uncat | |  |
| *Anampses meleagrides* | FMNH | | 124439 |
| *Anampses neoguinaicus* | FMNH | | 120173 |
| *Anampses twistii* | FMNH | | 119451 |
| *Austrolabrus maculatus* | FMNH | | 138043 |
| *Bodianus axillaris* | FMNH | | 124032 |
| *Bodianus bilunulatus* | Uncat | |  |
| *Bodianus diana* | FMNH | | 124487 |
| *Bodianus dictynna* | FMNH | | 124034 |
| *Bodianus loxozonus* | FMNH | | 112723 |
| *Bodianus mesothorax* | FMNH | | 126755 |
| *Bodianus pulchellus* | FMNH | | 73534 |
| *Bolbometopon muricatum* | FMNH | | 119522 |
| *Calotomus carolinus* | FMNH | | 123890 |
| *Calotomus japonicus* | FMNH | | 572478 |
| *Calotomus spinidens* | FMNH | | 110792 |
| *Centrolabrus exoletus* | BMNH | | 1975-8-18 |
| *Cetoscarus bicolor* | FMNH | | 110797 |
| Cheilinus_abudjubbe |  | |  |
| *Cheilinus bimaculatus* |  | |  |
| *Cheilinus chlorourus* | FMNH | | 120176 |
| *Cheilinus fasciatus* | FMNH | | 124051 |
| Cheilinus_lunulatus |  | |  |
| *Cheilinus oxycephalus* | FMNH | | 120182 |
| *Cheilinus trilobatus* | FMNH | | 126786 |
| *Cheilinus undulatus* | FMNH | | 110658 |
| *Cheilio inermis* | FMNH | | 126789 |
| *Chlorurus bleekeri* | FMNH | | 118756 |
| *Chlorurus bowersi* | FMNH | | 138383 |
| *Chlorurus capistratoides* | FMNH | | 123894 |
| *Chlorurus gibbus* | FMNH | | 110803 |
| *Chlorurus japanensis* | FMNH | | 118758 |
| *Chlorurus microrhinos* | FMNH | | 119524 |
| *Chlorurus oedema* | FMNH | | 110932 |
| *Chlorurus perspicillatus* | FMNH | | 73775 |
| Chlorurus_spilurus |  | |  |
| *Choerodon anchorago* | FMNH | | 119442 |
| *Choerodon oligacanthus* | FMNH | | 51758 |
| *Choerodon schoenleinii* | FMNH | | 110673 |
| *Choerodon zosterophorus* | FMNH | | 110674 |
| *Cirrhilabrus cyanopleura* | FMNH | | 126811 |
| *Cirrhilabrus exquisitus* | FMNH | | 126814 |
| *Cirrhilabrus punctatus* | FMNH | | 134111 |
| *Cirrhilabrus scottorum* | FMNH | | 127220 |
| *Cirrhilabrus temminckii* | FMNH | | 126819 |
| *Clepticus parrae* | FMNH | | 62916 |
| *Coris aygula* | FMNH | | 112777 |
| *Coris batuensis* | FMNH | | 110680 |
| *Coris dorsomacula* | FMNH | | 112778 |
| *Coris gaimard* | FMNH | | 110684 |
| *Coris julis* | FMNH | | 46108 |
| *Coris pictoides* | FMNH | | 126830 |
| *Coris venusta* | FMNH | | 128476 |
| *Cryptotomus roseus* | FMNH | | 65595 |
| *Ctenolabrus rupestris* | BMNH | | 1986-8-7 |
| *Cymolutes torquatus* | FMNH | | 110686 |
| *Decodon puellaris* | FMNH | | 66713 |
| *Diproctacanthus xanthurus* | FMNH | | 118887 |
| *Doratonotus megalepis* | FMNH | | 61535 |
| *Epibulus insidiator* | FMNH | | 86378 |
| *Eupetrichthys angustipes* | FMNH | | 138034 |
| *Gomphosus caeruleus* | FMNH | | 80054 |
| *Gomphosus varius* | FMNH | | 127214 |
| *Haletta semifasciata* | AMNH | | I.20180013 |
| *Halichoeres argus* | FMNH | | 124452 |
| *Halichoeres binotopsis* | FMNH | | 75982 |
| *Halichoeres bivittatus* | FMNH | | 45567 |
| *Halichoeres chloropterus* | FMNH | | 126858 |
| *Halichoeres chrysus* | FMNH | | 110699 |
| *Halichoeres dispilus* | FMNH | | 72294 |
| *Halichoeres garnoti* | FMNH | | 65314 |
| *Halichoeres hartzfeldii* | FMNH | | 110701 |
| *Halichoeres hortulanus* | FMNH | | 126864 |
| *Halichoeres leucurus* | FMNH | | 120159 |
| *Halichoeres maculipinna* | FMNH | | 65217 |
| *Halichoeres margaritaceus* | FMNH | | 112803 |
| *Halichoeres marginatus* | FMNH | | 124460 |
| *Halichoeres melanochir* | FMNH | | 126991 |
| *Halichoeres melanurus* | FMNH | | 124110 |
| *Halichoeres melasmapomus* | FMNH | | 119486 |
| *Halichoeres nebulosus* | FMNH | | 40651 |
| *Halichoeres podostigma* | FMNH | | 110709 |
| *Halichoeres poeyi* | FMNH | | 67745 |
| *Halichoeres prosopeion* | FMNH | | 120160 |
| *Halichoeres richmondi* | FMNH | | 124120 |
| *Halichoeres scapularis* | FMNH | | 118725 |
| *Halichoeres trimaculatus* | FMNH | | 112810 |
| *Hemigymnus fasciatus* | FMNH | | 127216 |
| *Hemigymnus melapterus* | FMNH | | 80132 |
| *Heteroscarus acroptilus* | AM | | 15751-005 |
| *Hipposcarus harid* | ANSP | | 103766 |
| *Hipposcarus longiceps* | FMNH | | 119526 |
| *Hologymnosus doliatus* | FMNH | | 121072 |
| *Iniistius bimaculatus* | FMNH | | 127213 |
| *Iniistius dea* | FMNH | | 57355 |
| *Iniistius pavo* | UWFC | | 7203 |
| *Iniistius pentadactylus* | FMNH | | 47707 |
| *Iniistius umbrilatus* | FMNH | | 128485 |
| *Labrichthys unilineatus* | FMNH | | 120192 |
| *Labroides pectoralis* | FMNH | | 119497 |
| *Labropsis australis* | FMNH | | 127211 |
| *Labrus merula* | FMNH | | 109161 |
| *Labrus mixtus* | BMNH | | FS492 |
| *Labrus viridis* | FMNH | | 109162 |
| *Lachnolaimus maximus* | ANSP | | 49141 |
| *Leptoscarus vaigiensis* | FMNH | | 110821 |
| *Macropharyngodon geoffroy* | FMNH | | 63593 |
| *Macropharyngodon meleagris* | FMNH | | 124602 |
| *Macropharyngodon negrosensis* | Uncat | |  |
| *Malapterus reticulatus* | FMNH | | 107424 |
| *Neodax balteatus* | AM | | I.20179-015 |
| *Nicholsina usta* | USNM | | 202348.5062 |
| *Notolabrus gymnogenis* | FMNH | | 135921 |
| *Novaculichthys taeniourus* | FMNH | | 118735 |
| *Novaculoides macrolepidotus* | FMNH | | 110729 |
| *Odax cyanoallix* | AMNH | | 17019008 |
| *Odax pullus* | AMNH | | 123364001 |
| *Olisthops cyanomelas* | AMNH | | I.17019008 |
| *Ophthalmolepis lineolata* | FMNH | | 137991 |
| *Oxycheilinus arenatus* | BPBM | | 24119 |
| *Oxycheilinus celebicus* | FMNH | | 127140 |
| *Oxycheilinus digramma* | FMNH | | 124596 |
| Oxycheilinus_nigromarginatus |  | |  |
| *Oxycheilinus orientalis* | FMNH | | 127162 |
| Oxycheilinus_unifasciatus |  | |  |
| *Oxyjulis californica* | FMNH | | 77933 |
| *Paracheilinus filamentosus* | FMNH | | 127167 |
| *Pictilabrus laticlavius* | FMNH | | 137988 |
| *Pseudocheilinops ataenia* | FMNH | | 124174 |
| *Pseudocheilinus evanidus* | FMNH | | 130899 |
| *Pseudocheilinus hexataenia* | FMNH | | 112884 |
| *Pseudocheilinus octotaenia* | FMNH | | 118982 |
| *Pseudocoris yamashiroi* | FMNH | | 119504 |
| *Pseudodax moluccanus* | FMNH | | 119120 |
| *Pseudojuloides cerasinus* | FMNH | | 127186 |
| *Pseudolabrus guentheri* | FMNH | | 135927 |
| *Pseudolabrus luculentus* | FMNH | | 137990 |
| *Pteragogus cryptus* | FMNH | | 127188 |
| *Scarus chameleon* | FMNH | | 110833 |
| *Scarus coelestinus* | ANSP | | 75173 |
| *Scarus coeruleus* | FMNH | | 46860 |
| *Scarus dimidiatus* | FMNH | | 127258 |
| *Scarus dubius* | FMNH | | 49154 |
| *Scarus festivus* | FMNH | | 118765 |
| *Scarus flavipectoralis* | FMNH | | 123906 |
| *Scarus forsteni* | FMNH | | 127261 |
| *Scarus frenatus* | ANSP | | 151966 |
| *Scarus globiceps* | ANSP | | 9279 |
| *Scarus guacamaia* | JFBM | | 29376 |
| *Scarus hypselopterus* | FMNH | | 110849 |
| *Scarus maculipinna* | FMNH | | 117286 |
| *Scarus niger* | FMNH | | 127266 |
| *Scarus oviceps* | FMNH | | 118773 |
| *Scarus prasiognathos* | ANSP | | 151958 |
| *Scarus psittacus* | FMNH | | 110857 |
| *Scarus quoyi* | FMNH | | 127343 |
| *Scarus rubroviolaceus* | ANSP | | 51097 |
| *Scarus russelii* | ANSP | | 103714 |
| *Scarus schlegeli* | ANSP | | 122616 |
| *Scarus spinus* | FMNH | | 119521 |
| *Scarus taeniopterus* | ANSP | | 9290 |
| *Scarus tricolor* | FMNH | | 118780 |
| *Scarus viridifucatus* | ANSP | | 109275 |
| *Semicossyphus pulcher* | FMNH | | 62769 |
| *Siphonognathus argyrophanes* | AMNH | | 120180014 |
| *Siphonognathus radiatus* | AMNH | | I.200228008 |
| *Sparisoma aurofrenatum* | FMNH | | 108733 |
| *Sparisoma axillare* | FMNH | | 74653 |
| *Sparisoma choati* | UF | | 179646 |
| *Sparisoma chrysopterum* | FMNH | | 67987 |
| *Sparisoma cretense* | ANSP | | 9281 |
| *Sparisoma radians* | FMNH | | 5391 |
| *Sparisoma rubripinne* | ANSP | | 9294 |
| *Sparisoma viride* | ANSP | | 75014 |
| *Stethojulis balteata* | FMNH | | 80145 |
| *Stethojulis bandanensis* | FMNH | | 110746 |
| *Stethojulis interrupta* | FMNH | | 124466 |
| *Suezichthys gracilis* | FMNH | | 127196 |
| *Symphodus mediterraneus* | FMNH | | 109164 |
| *Symphodus ocellatus* | FMNH | | 63840 |
| *Symphodus rostratus* | FMNH | | 109168 |
| *Symphodus tinca* | FMNH | | 46103 |
| *Tautoga onitis* | FMNH | | 5857 |
| *Tautogolabrus adspersus* | JFBM | | 14932 |
| *Terelabrus dewapyle* | FMNH | | 133681 |
| *Thalassoma amblycephalum* | FMNH | | 130928 |
| *Thalassoma ballieui* | FMNH | | 129436 |
| *Thalassoma bifasciatum* | FMNH | | 108729 |
| *Thalassoma cupido* | FMNH | | 89117 |
| *Thalassoma duperrey* | FMNH | | 128475 |
| *Thalassoma hardwicke* | FMNH | | 127202 |
| *Thalassoma lunare* | FMNH | | 120214 |
| *Thalassoma lutescens* | FMNH | | 121076 |
| *Thalassoma quinquevittatum* | FMNH | | 127208 |
| *Wetmorella nigropinnata* | FMNH | | 130978 |
| *Xenojulis margaritaceus* | FMNH | | 110771 |
| *Xiphocheilus typus* | FMNH | | 110772 |
| *Xyrichtys martinicensis* | JFBM | | 19343 |
| *Xyrichtys novacula* | FMNH | | 46615 |

**Table S3.** Descriptions of homologous anatomical landmarks and curves for sliding semi-landmarks adapted from LaRouche et al. 2022. The three points added to this study are bolded (192-194).

| **Homologous anatomical landmarks** | |
| --- | --- |
| **Landmark #** | **Definition** |
| 1 | Most distal point of first tooth on premaxilla |
| 2 | Base of first tooth |
| 3 | Most distal point of ascending process of premaxilla |
| 4 | Proximal vertex between ascending and descending processes of premaxilla |
| 5 | Most distal point of descending process of premaxilla |
| 6 | Most distal point of first tooth on dentary |
| 7 | Posterior-most point on dentary flange |
| 8 | Ventral-most point of mental symphysis on dentary |
| 9 | Antero-distal-most point of articular/angular |
| 10 | Distal-most point of coronoid process |
| 11 | Ventral, posterior-most point of dentary |
| 12 | Center of jaw joint on quadrate |
| 13 | Posterior-most point on retroarticular |
| 14 | Most anterior point of vomer |
| 15 | Left, distal-most point on anterior of vomer (used to approximate width of vomer) |
| 16 | Proximal lachrymal-lateral ethmoid margin |
| 17 | Proximal lateral ethmoid-parasphenoid margin |
| 18 | Dorsal-most point of lachrymal |
| 19 | Proximal-most point of lateral ethmoid frontal margin |
| 20 | Origin of supraoccipital |
| 21 | Posterior frontal sub-orbital margin |
| 22 | Pterosphenoid-parasphenoid margin |
| 23 | Mid-point parasphenoid-basioccipital margin |
| 24 | Prootic foramen |
| 25 | Frontal parietal margin |
| 26 | Posterior-most point of supraoccipital |
| 27 | Ventral epiotic-post temporal margin |
| 28 | Distal-most dorsal point of basioccipital |
| 29 | Pterotic-post temporal margin |
| 30 | Distal-most ventral point of basioccipital |
| 31 | Anterior most point of lower pharyngeal jaw plate |
| 32 | Descending process of lower pharyngeal jaw plate |
| 33 | Lateral-central most point of pharyngeal tooth plate |
| 34 | Ventral base of descending process of pharyngeal tooth plate |
| 35 | Lateral wing of pharyngeal tooth plate |
| 36 | Posterior center-most point of pharyngeal tooth plate |
| 87 | Distal,dorsal anterior margin of maxilla |
| 88 | Distal,dorsal posterior margin of maxilla |
| 89 | Distal, ventral anterior margin of maxilla |
| 90 | Distal, ventral posterior margin of maxilla |
| 91 | Proximal,dorsal anterior margin of maxilla |
| 92 | Proximal,dorsal posterior margin of maxilla |
| 93 | Anterior-most point of the proximal descending process of maxilla |
| 94 | anterior-most pont of contact on the distal face of the hyomandibula between hyomandibula and sphenotic |
| 95 | posterior-most point of contact on the distal face of the hyomandibula between the hyomandibula and the pterotic |
| 96 | Distal-most point of lateral projection of hyomandibula |
| 97 | Anterior-ventral most point of hyomandibula |
| 98 | Ventral-most point of hyomandibula |
| 99 | Anteriror, proximal-most point of nasal |
| 100 | Anteriror, distal-most point of nasal |
| 101 | Posterior, proximal-most point of nasal |
| 102 | Posterior, distal-most point of nasal |
| 150 | Dorsal, anterior-most point of urohyal |
| 151 | Ventral anterior-most point of urohyal |
| 152 | Dorsal, posterior-most point of urohyal |
| 153 | Ventral, posterior-most point of urohyal |
| 154 | Anteriror-most point of ceratohyal |
| 155 | Interior-ridge of ceratohyal |
| 156 | Dorsal ceratohyal-epihyal margin |
| 157 | Posterior-most point of epihyal |
| 158 | Ventral ceratohyal-epihyal margin |
| 179 | left outside angle of preoperculum |
| 180 | left anterior tip of preoperculum |
| 181 | left interopercle-opercle joint |
| 182 | left joint between preoperculum and neurocranium |
| 183 | left joint between hyomandibula and operculum |
| 184 | left anterior tip of palatine |
| 185 | left dorsal point on curve of palatine |
| 186 | left ventral tip of palatine at pterygoid joint |
| 187 | left dorsal tip of palatine at pterygoid joint |
| 188 | anterior tip of hypohyal |
| 189 | posterior tip of hypohyal |
| 190 | joint between hypohyal and ceratohyal |
| 191 | left dorsal tip of interhyal |
| **192** | **left ventral tip of pectoral girdle** |
| **193** | **left joint between cleithrum and supracleithrum** |
| **194** | **left joint between supracleithrum and posttemporal** |
| 195 | left side of joint between upper pharyngeal jaws |
| 196 | left dorsal point of upper pharyngeal jaws |
| 197 | left ventral point of upper pharyngeal jaws |
| 198 | left lateral point on upper pharyngeal jaws |
| 199 | left upper pharyngeal jaw joint |
| 200 | left uppermost tip of upper pharyngeal jaw |
| **Homologous curves for the positioning of sliding semi-landmarks** | |
| **Landmark #** | **Definition** |
| 37-47 | Curve 1: Ascending process of premaxilla, between landmarks 2 and 3 |
| 48-51 | Curve 2: Ventral margin of dentary, between landmarks 8 and 11 |
| 52-62 | Curve 3: Ventral margin of parasphenoid, between landmarks 14 and 23 |
| 63-77 | Curve 4: Supraoccipital crest, between landmarks 20 and 26 |
| 78-86 | Curve 5: Orbital margin, between landmarks 18 and 21 |
| 103-106 | Curve 6: Angular ascending process, between landmarks 10 and 12 |
| 107-110 | Curve 7: Angular lateral process, between landmarks 9 and 12 |
| 111-115 | Curve 8: Lateral arm of lower pharyngeal tooth plate, between landmarks 34 and 35 |
| 116-120 | Curve9: Descending process of lower pharyngeal tooth plate, between landmarks 34 and 32 |
| 121-126 | Curve 10: Descending arm of premaxilla, between landmarks 4 and 5 |
| 127-131 | Curve 11: Lateral arm of premaxilla, between landmarks 2 and 5 |
| 132-135 | Curve 12: Anterior face of hyomandibula, between landmarks 94 and 97 |
| 136-139 | Curve 13: Dorsal surface of hyomandibula, between landmarks 94 and 95 |
| 140-143 | Curve 14: Posterior face of hyomandibula, between landmarks 96 and 98 |
| 144-146 | Curve 15: Nasal interior surface, between landmarks 99 and 101 |
| 147-149 | Curve 16: Nasal outer edge, between landmarks 100 and 102 |
| 159-163 | Curve 17: Urohyal dorsal edge, between landmarks 150 and 152 |
| 164-168 | Curve 18: Urohyal ventral edge, between landmarks 151 and 153 |
| 169-173 | Curve 19: Ceratohyal dorsal ridge, between landmarks 154 and 156 |
| 174-178 | Curve 20: Ceratohyal ventral ridge, between landmarks 154 and 158 |

Figure S1: Taken with permission from LaRouche et al. 2022. 3D model of the landmark (blue dots) and semi-landmark (red dot) positions. Illustrated on a *Halichoeres bivittatus* specimen.

**
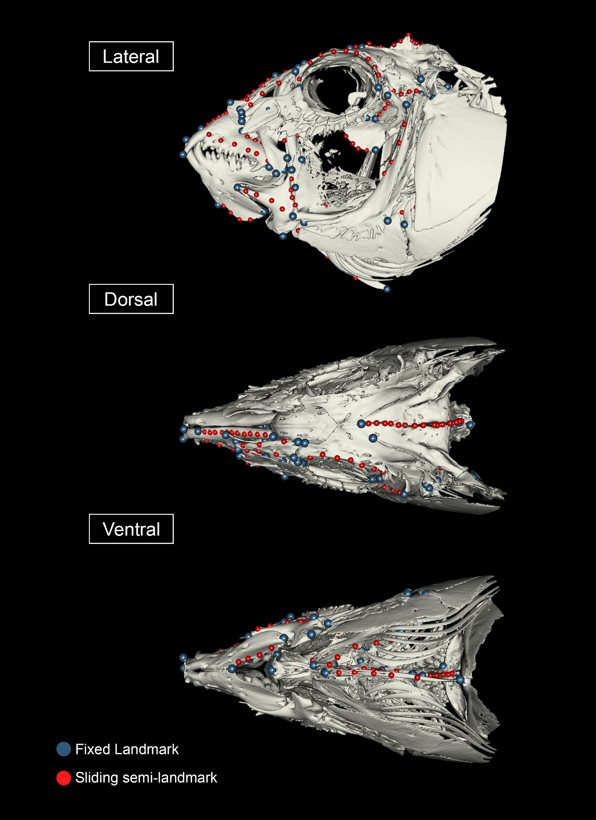
**
